# Robust estimation of quantitative perfusion from multi‐phase pseudo‐continuous arterial spin labeling

**DOI:** 10.1002/mrm.27965

**Published:** 2019-08-20

**Authors:** Y. Msayib, M. Craig, M. A. Simard, J. R. Larkin, D. D. Shin, T. T. Liu, N. R. Sibson, T. W. Okell, M. A. Chappell

**Affiliations:** ^1^ Department of Engineering Science Institute of Biomedical Engineering University of Oxford Oxford United Kingdom; ^2^ Department of Oncology Cancer Research UK and Medical Research Council (CRUK/MRC) Oxford Institute for Radiation Oncology University of Oxford Oxford United Kingdom; ^3^ GE Healthcare Menlo Park California; ^4^ Center for Functional MRI University of California San Diego California; ^5^ Wellcome Centre for Integrative Neuroimaging FMRIB Nuffield Department of Clinical Neurosciences University of Oxford Oxford United Kingdom

**Keywords:** estimation bias, multi‐phase pseudo‐continuous arterial spin labeling, perfusion quantification, supervoxel clustering

## Abstract

**Purpose:**

Multi‐phase PCASL has been proposed as a means to achieve accurate perfusion quantification that is robust to imperfect shim in the labeling plane. However, there exists a bias in the estimation process that is a function of noise in the data. In this work, this bias is characterized and then addressed in animal and human data.

**Methods:**

The proposed algorithm to overcome bias uses the initial biased voxel‐wise estimate of phase tracking error to cluster regions with different off‐resonance phase shifts, from which a high‐SNR estimate of regional phase offset is derived. Simulations were used to predict the bias expected at typical SNR. Multi‐phase PCASL in 3 rat strains (*n* = 21) at 9.4 T was considered, along with 20 human subjects previously imaged using ASL at 3 T. The algorithm was extended to include estimation of arterial blood flow velocity.

**Results:**

Based on simulations, a perfusion estimation bias of 6‐8% was expected using 8‐phase data at typical SNR. This bias was eliminated when a high‐precision estimate of phase error was available. In the preclinical data, the bias‐corrected measure of perfusion (107 ± 14 mL/100g/min) was lower than the standard analysis (116 ± 14 mL/100g/min), corresponding to a mean observed bias across strains of 8.0%. In the human data, bias correction resulted in a 15% decrease in the estimate of perfusion.

**Conclusions:**

Using a retrospective algorithmic approach, it was possible to exploit common information found in multiple voxels within a whole region of the brain, offering superior SNR and thus overcoming the bias in perfusion quantification from multi‐phase PCASL.

## 1 INTRODUCTION

Pseudo‐continuous labeling is now widely accepted as the preferred scheme for ASL perfusion imaging due to superior signal‐to‐noise ratio (SNR) over pulsed variants. A shortcoming of PCASL, however, is the sensitivity of labeling to phase mismatch due to the presence of inhomogeneous magnetic fields and variations in arterial blood flow velocity in the labeling plane. Off‐resonance and flow velocity effects can lead to inefficient labeling, resulting, in the worst case, in the loss of perfusion information in a whole perfusion territory, or at least variations in labeling efficiency for blood destined for different regions of the brain, not routinely accounted for in quantification methods.^1^ Phase mismatch can be reduced by effective shimming in the labeling region, something that is not necessarily routinely performed as shimming is more commonly applied only to the imaging region. Since phase mismatch is directly related to field homogeneity it is particularly acute at higher field strength and thus of particular relevance to the use of PCASL at 7 T in humans, as well as the use at even higher fields pre‐clinically in small animal studies.

Methods have been proposed to correct for the effect using a $B_{0}$ field map,^1^ or to measure the labeling efficiency in individuals.^2^ A pre‐scan procedure has also been described where off‐resonance effects at vessel locations are measured and then compensated for during labeling by appropriately adjusting the RF phase increment.^3^ More recently a method to measure inversion efficiency in individual arteries using a separate short ASL scan^4^ has been presented, but only demonstrated for the internal carotid arteries. These methods permit post hoc correction for phase mismatch, along with other sources of variation in inversion efficiency provided inversion efficiency is measured directly. However, these methods either provide a global correction for the whole brain, or artery‐specific information that cannot simply be applied to the data without separate knowledge of the perfusion territories.

An alternative solution is to acquire data at a range of phase offsets and then use subsequent model fitting to extract the magnitude of the signal change associated with the delivery of labeled blood, taking into account the variation in labeling efficiency with phase offset, which can then be used to reconstruct the perfusion image.^5^ This strategy offers an overall lower temporal efficiency than ideal PCASL labeling because data are acquired in a range of suboptimal partial control or label conditions. However, this scheme can be applied voxel‐wise to correct for the effects of phase mismatch that apply to the labeled blood‐water that has supplied each voxel. Alternatively, the information gained from a multi‐phase pre‐scan can be used to adjust the labeling for a subsequent PCASL acquisition.^6^ In principle, multi‐phase PCASL might additionally allow for the estimation of flow velocity in the labeled arteries through knowledge of the relationship between signal at different phase offsets and flow velocity, although SNR limitations prevented this from being sufficiently robust at 3 T in the study of Refs. [6, 7].

The basic approach to multi‐phase PCASL perfusion quantification, where multi‐phase data are fitted to the modified Fermi function,^5^ has been found to be limited by systematic overestimation in a manner correlated with SNR.^8^, ^9^ Bias in quantification using the basic approach is due to the limited number of sampling points (RF phases) available, from which estimation of multiple model parameters is sought,^8^ and is consistent with studies that have been performed on the fitting of a sinusoidal profile to data with additive noise and a limited number of samples.^10^ The bias is primarily attributable to the fact that physiological and motion‐induced noise tends to cancel out in the conventional tag‐control subtraction and averaging operations, while, in the multi‐parameter model fitting of multi‐phase data, the noise is inherent in the ASL signal, thereby biasing the estimate of the scaling term. While tag‐control ASL is not subject to such overestimation bias, it is, however, confounded by inversion efficiency, which is precisely the confound that MP PCASL is robust to by virtue of estimating phase offset. This study sets out the technical details of a quantification scheme that incorporates robust detection of phase offset information from perfusion images by exploiting recent machine learning representations of data in the form of supervoxels and clustering. The technical issues arising from low‐SNR perfusion estimation are illustrated, and a robust solution is presented. Further to this, we examine whether the method described in this work would permit flow velocity estimation in feeding arteries.

## 2 THEORY

### 2.1 Modified Fermi function

Multi‐phase PCASL involves the acquisition of images over a range of phase offsets (θ), where pseudo‐continuous labeling has been applied in a plane through the neck intersecting the major arteries feeding the brain. The resulting images contain a common static tissue contribution, $M_{s}$, plus a modulated contribution, Δ*M*, from partially‐labeled blood. The multi‐phase PCASL signal, shifted by an off‐resonance phase term ϕ, can be approximated by a modified Fermi function^5^:

(1) $$f\left(\theta -\phi \right)=M_{s}-\Delta M\left(\frac{2}{1+e^{\left(|\theta -\phi |-\alpha \right)/\beta }}-1\right)$$

where the parameters α and β are shape parameters of the modified Fermi function. At 3T in humans, shape parameter values were found to be α = 54 and β = 13.^5^ The preclinical study of Ref. [8] found α = 70 and β = 19 for rats. By voxel‐wise fitting of this function to the phase offset data the amplitude can be extracted, providing a measure of optimal label‐control difference, Δ*M*. Δ*M* is then directly used to calculate quantitative perfusion in mL/100g/min.

#### 2.1.1 Estimation of flow velocity

In reality the modified Fermi function is only an approximation to the real variation with phase offset. A more accurate model can be derived by modeling the flow and interaction with the PCASL labeling pulses (Supporting Information Figure S1).^11^, ^12^ This results in a numerical description of the profile that can be fit to the data and allows the flow velocities of the blood in the arteries to be taken into account. The subtle effect of flow velocity means that relatively high SNR is needed for estimation of this parameter, hence it has thus far not been successful for voxel‐wise estimation from multi‐phase PCASL perfusion imaging at 3 T in humans.^13^ However, this has been used in a technique for vessel‐encoded PCASL analysis where parameter estimation was effectively performed across a whole perfusion territory, thus increasing the SNR by averaging of multiple voxels.^11^, ^14^

## 3 METHODS

### 3.1 Simulations of perfusion quantification bias in multi‐phase PCASL

The simulation study was carried out using MATLAB (MathWorks Inc., Natick, Massachusetts). To investigate bias in the estimation of Δ*M* at low SNR, data were simulated according to the modified Fermi function in Equation 1 at 8 phase offsets equally spaced across $360^{\circ }$, adding white noise at varying SNR (20 to 100). SNR was defined relative to the offset, i.e. static tissue magnitude, $M_{s}$, with multiple instances of noise generated at each SNR. The modified Fermi function was then fitted to the noisy simulated data, with 3 variables being estimated: magnitude Δ*M*, phase offset ϕ (incorporated by fitting for *f*(θ−ϕ)), and static tissue magnetization offset $M_{s}$, using a variational Bayesian model fitting routine.^15^, ^16^ A low‐precision prior was used for each variable to reflect a high level of uncertainty with respect to the true values. The dependence of bias on the ratio of invertible blood to static blood, $\frac{\Delta M}{M_{s}}$, was found in a similar way at each SNR. Δ*M* was varied from 1% to 32% of $M_{s}$, with $M_{s}\;=\;1000$. To observe the effect that a good estimate of ϕ has on the systematic bias at low SNR, the SNR study was repeated using a fixed value of ϕ (achieved using a high‐precision prior, equivalent to a constant), which effectively reduced the number of unknown parameters to be fitted for to 2 parameters (Δ*M* and $M_{s}$).

### 3.2 Preclinical data acquisition

The imaging and data acquisition protocols used for the animal data in this study are detailed in Ref. [8]. Briefly, multi‐phase PCASL was acquired in isoflurane anesthetized female Wistar, Sprague Dawley (SD), and Berlin Druckery *IX* (BDIX) rats (*n* = 3 per strain) at 9.4 T (Agilent). The sequence used 8 equally spaced phase angles ranging from $0^{\circ }$ to $315^{\circ }$, with an optimal label duration of 1400 ms, a post‐label delay of 550 ms, and labeling plane positioned perpendicular to the brain feeding arteries ($45^{\circ }$ angle) and directly behind the medulla oblongata.^8^ The RF labeling pulse parameters were: labeling gradient amplitude: 16.3 mT/m, average gradient amplitude: 0.8 mT/m, RF pulse interval: 1200 μs, RF pulse width: 600 μs (Hanning‐shaped pulses), with an RF flip angle of $40^{\circ }$. A multi‐slice single‐shot spin echo EPI sequence was used for the imaging readout, with a 32 × 32 mm FOV (64 × 64 matrix, thickness of 1 mm, 10 slices), *TE* = 28.7 ms. Proton density (PD) calibration images (*TR* = 7.6 s) were acquired for each animal by omitting labeling pulses, using both the surface receive array and volume coils. Quantitative $T_{1}$ maps were obtained for all animals using inversion recovery (*TI* varied in 9 logarithmic steps from 13 ms to 8 s), as were quantitative $T_{2}$ maps (multi‐echo approach where *TE* was varied in 10 logarithmic steps from 30 to 160 ms, *TR* = 10 s). $T_{1}$ and $T_{2}$ of re‐oxygenated post‐mortem blood were determined at $37^{\circ }C$. Measurements of carotid blood flow velocity were made for each rat strain using Doppler ultrasound (*n* = 3 per strain), done with different rats to those used for MR imaging. Further details concerning the imaging and data acquisition protocols used for the animal data in this study can be found in Ref. [8].

### 3.3 Human MR imaging

Twenty healthy subject MRI datasets were randomly selected from the CBFBIRN database for use in this study (11 female, median age: 76 years). The datasets were acquired on a 3 T GE Discovery MR750 MRI scanner with a body transmit coil and an 8‐channel receive‐only head coil. Each dataset comprised a high‐resolution structural scan, a PD‐weighted CSF calibration scan, and a multi‐phase ASL scan, as follows. The structural scan was acquired using a magnetization‐prepared 3*D* fast spoiled gradient recalled (FSPGR) sequence, with parameters: 1 mm isotropic resolution, 172 sagittal slices, *TI* = 600 ms, *TR* = 7.9 ms, *TE* = 3.1 ms, flip angle of $8^{\circ }$, and a parallel imaging acceleration factor of 2 (scan time: 3 min 45 s). The CSF calibration scan was obtained using a spiral readout with *TR* = 4 *s* and *TE* = 3.4 ms, and comprised 9 $90^{\circ }$ excitation pulses which were turned off for the first 8 repetitions to generate PD‐weighted contrast (scan time: 36 s). For the MP‐PCASL scans, imaging was done at 8 RF phase offsets (evenly spaced from $0^{\circ }$ to $315^{\circ }$) with the following parameters: *TR* = 4200 ms, *TE* = 3.3 ms, *FOV* = 240 × 240 mm, matrix size 64 × 64, 20 axial slices, and voxel dimensions of 3.75 × 3.75 × 5.95 mm. Eight repetitions were acquired at each of the 8 phase offsets, and the total scan time was 4 min 30 s. A single‐shot spiral gradient echo readout was used for each axial slice. The PCASL labeling parameters of the MP‐PCASL volumes were: labeling period of 2000 ms, post‐labeling delay of 1600 ms, labeling gradient amplitude: 1.6 mT/m, average gradient amplitude: 0.06 mT/m, RF pulse interval: 998 μs, RF pulse width: 375 μs (Hanning‐shaped pulses), RF flip angle of $28.2^{\circ }$ (corresponding to a maximum $B_{1}$ amplitude of 10 μT), with background suppression turned off.

### 3.4 Multi‐stage solution for bias‐corrected perfusion quantification

The fitting of the modified Fermi function to multi‐phase PCASL data involves the estimation of 3 parameters in Equation (1): the amplitude of the modified Fermi function, Δ*M*, which provides a measure of the perfusion in a voxel as would be measured with ideal label and control subtraction; an offset ($M_{s}$) due to a static tissue contribution; and ϕ, the phase offset associated with the artery in which labeling was performed. To get an unbiased estimate irrespective of SNR, it is only necessary to know 1 of 3 parameters.^8^ In other words, if the phase offset ϕ is known, then an unbiased estimate of Δ*M* can be obtained by fitting of the modified Fermi function. The strategy adopted in our method is to robustly estimate the off‐resonance phase term ϕ from a larger ROI, in which multiple voxels are first averaged, followed by voxel‐wise perfusion estimation with the off‐resonance term fixed. The key concept behind phase estimation is that the phase parameter is specific to the feeding arteries, and thus is common across a large number of voxels. Regions of common phase offset are deduced by first fitting the modified Fermi function to the data using all 3 parameters, resulting in biased parameter estimates. Even though, in the resulting parameter maps, the absolute values of the parameters may be incorrect, *differences* in phase offsets across vascular territories are preserved, allowing appropriate regions of interest to be identified according to regions with different phase (and thus potentially a different feeding artery). Averaging of data within an ROI results in data with a higher SNR, and therefore reduced bias when estimating the phase parameter.

**Figure 1:**
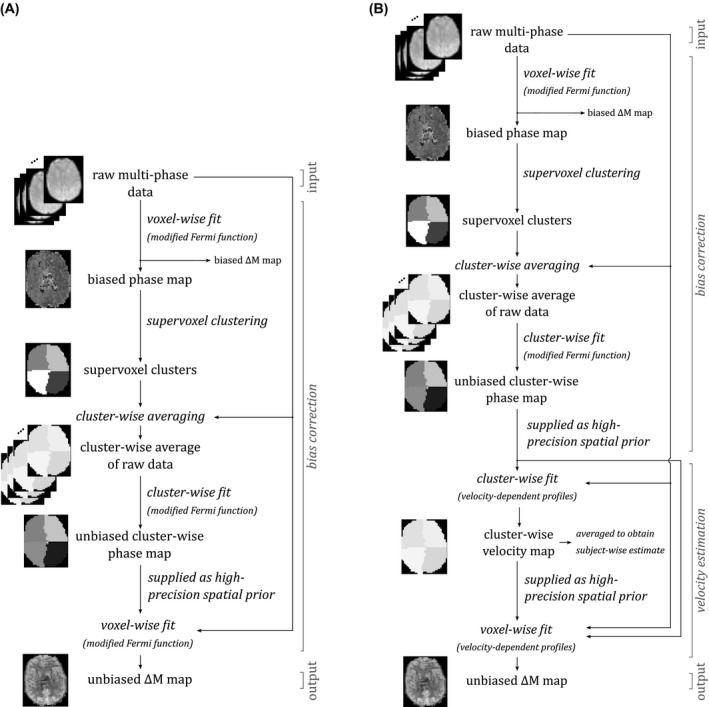
A, Multi‐stage procedure proposed to overcome the bias observed in the model‐fitting procedure for multi‐phase PCASL data. B, Incorporating velocity estimation into the multi‐stage bias correction procedure. Inputs and outputs are denoted by normal font, and processes are denoted by italicized text

**Figure 2:**
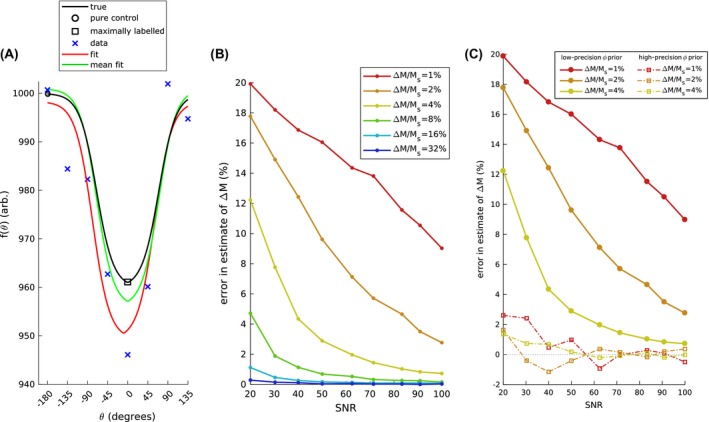
A, Example of simulated multiphase data including the underlying true function, a point representing a pure control ($f\;=\;M_{s}\;+\;\Delta M$), a maximally‐labeled point ($f\;=\;M_{s}-\Delta M$), a noisy realization of the true function (*SNR* = 40), the model fit, and the function estimated when taking the mean over multiple realizations. B, The effect of SNR on estimation of Δ*M*, where the error is relative to the ground truth. Δ*M* is expressed as a fraction of static tissue magnetization. C, The effect of SNR on estimation of Δ*M* (and hence perfusion), shown 2 for cases: the solid lines are where a low‐precision prior was used for estimating ϕ, and the dashed lines are where a high‐precision prior was used for estimating ϕ. Here magnitude is relative to the offset (static tissue) parameter

Figure 1A illustrates this proposed multi‐stage procedure for overcoming the bias observed in the model‐fitting procedure for multi‐phase PCASL data (an example of the bias is shown in Figure 2A). The algorithm proceeds as follows: (1) an initial voxel‐wise fit to produce biased maps of the parameters; (2) a supervoxel based clustering procedure^17^ on the biased phase map to identify regions of common phase; (3) average series in each cluster on which (4) model‐fitting is performed, (5) additional voxel‐wise model‐fitting with the phase parameter fixed by the appropriate value from the cluster analysis.

To robustly define ROIs from the resulting phase offset map, a supervoxel algorithm is applied to group regions of common phase offset, with the number of classes set to 4 (representing 4 feeding arteries, with supervoxel parameters: compactness = 0.1, smoothing = 0.8).^17^ With the ROIs defined the multi‐phase data in each ROI is averaged and model fitting performed. The resulting phase offset values are then used as fixed estimates in a final voxel‐wise model fitting of the data. The parameters used for the animal data were α = 70, β = 19,^8^ and α = 54, β = 13 for the human data.^5^ Model fitting was performed using the using a variational Bayesian non‐linear model inference method^15^ implemented in the FABBER program that is distributed as part of the BASIL toolbox (http://www.fsl.fmrib.ox.ac.uk/fsl/fslwiki/BASIL) for ASL perfusion quantification in the FMRIB Software Library.^18^, ^19^, ^20^ The multi‐stage method, including model‐fitting, supervoxel analysis and clustering was implemented in *Quantiphyse* and is made available for download at http://www.quantiphyse.org.

### 3.5 Incorporating velocity estimation into the multi‐stage solution

The modified Fermi function is only an approximation to the variation in signal inversion with phase offset. Simulations of profiles based on flow velocity might be more accurate in practice, hence their inclusion in the multi‐stage solution was also explored. The method outlined above was modified to estimate flow velocity in feeding arteries by replacing the Fermi function in the final stage of fitting, with a model of the expected flow profiles (Figure 1B). The expected flow profiles were generated by simulation of the Bloch Equations using the hard pulse approximation, where the effects of $T_{1}$ relaxation were neglected and a $T_{2}$ of 200 ms was assumed for arterial blood.^5^ The pulse and gradient parameters adopted in the simulations were identical to those used in data acquisition, except in the case of the preclinical data where a mean gradient amplitude of 1.62 mT/m was used instead of 0.81 mT/m as the former provided a better fit empirically. Profiles were generated for a range of mean flow speeds (5, 10, 20, 30, 40, 50, and 60 cm/s) (shown in Supporting Information Figure S1). Poiseuille flow was assumed and the parabolic velocity profile for a given mean flow speed was discretized into 6 segments; the simulation results from the 6 constituent velocities were averaged, weighted by annular area of each radial discretization step. Each simulated inversion profile was comprised of 200 phase offsets between ±*π*, forming a lookup table of blood magnetization as a function of phase offset and mean flow velocity (200 data points × 6 mean flow speeds). This matrix of solutions was loaded into the FABBER program and linear interpolation used to evaluate the model at values between those simulated. Velocity estimates were derived from the data as follows. The velocity estimates from the data were done through a model fitting scheme that identifies the Bloch line shape that best fits the data, where the line shapes are chosen from the table of pre‐computed line shapes that each encode a different velocity. Each pre‐computed line shape is associated with a known velocity, hence when a line shape is chosen that fits the data well, a value for velocity is obtained with it. The modified Fermi function was fitted for in the first stage of the data analysis, and then after ROI generation, in the ROIs. The phase offset for each ROI was then fixed for the final analysis, where the Fermi function was swapped for the lookup table of velocity‐dependent profiles, used to derive the final perfusion images.

### 3.6 Calibration of quantitative perfusion maps

After the Δ*M* maps had been obtained using the standard method and using the multi‐stage solution, they were used as label‐control difference images from which perfusion was quantified. For the animal data, absolute perfusion values were derived by estimating the equilibrium magnetization in the striatum and converting to the equivalent value in blood based on the partition co‐efficient.^8^ A coil sensitivity correction map was derived from the ratio of the surface receive array and volume coil control images. Relaxation parameters used in quantification are detailed in Ref. [8].

For the human data, motion correction was applied to across the MP‐PCASL acquisitions using FSL MCFLIRT, which rigidly registered the 64 volumes to the first time point.^21^ Brain masks were obtained for the ASL, PD‐weighted, and anatomical scans using the FSL Brain Extraction Tool.^22^ Perfusion measures were evaluated in ASL space within a gray matter ROI. The gray matter ROI was obtained by first generating a gray matter partial volume estimate (PVE) map in anatomical space using FSL FAST,^23^ which was transformed to ASL space and binarised using a threshold of 0.7. Registration from anatomical to functional space was done by obtaining the affine transform from functional to anatomical space, and then applying the inverse of that transform to the anatomical image, with a super‐sampling factor of 4. Calibration of the perfusion maps was done using the reference region method,^24^ where CSF magnetization was estimated from the PD‐weighted scan. Coil sensitivity correction utilized the estimated bias field map from FSL FAST. The analysis pipeline for converting perfusion‐weighted images (i.e. Δ*M* maps) to quantitative perfusion maps was invoked using the Oxford ASL tool in BASIL (http://fsl.fmrib.ox.ac.uk/fsl/fslwiki/oxford_asl).^15^, ^25^

### 3.7 Analysis of imaging data

All analysis was done in the ASL space of the data, using Python libraries and MATLAB. Goodness of fit between the acquired data and the model fits was evaluated using the coefficient of determination ($R^{2}$). The mean perfusion measures from standard analysis and bias‐corrected were compared for each rat strain. The mean ratio $\frac{\Delta M}{M_{s}}$ was found in animals by voxel‐wise averaging of the ratio of the model‐fitted parameters Δ*M* and $M_{s}$ (within the whole brain). The mean SNR was found by voxel‐wise averaging of the ratio of estimated $M_{s}$ to noise standard deviation, where the latter was estimated for each voxel's time series as part of the FABBER model fitting stage.

The mean distance between the 4 phase offsets within a subject was used as a measure of intra‐subject consistency of phase offset. For this measure, averaging was done over unique paired combinations from a subject's 4 clusters: $\frac{1}{6}\cdot \left(|\phi_{1}-\phi_{2}\left|+\right|\phi_{1}-\phi_{3}\left|+\right|\phi_{1}-\phi_{4}\left|+\right|\phi_{2}-\phi_{3}\left|+\right|\phi_{2}-\phi_{4}\left|+\right|\phi_{3}-\phi_{4}|\right)$. The mean difference in phase offset between subjects was used as an inter‐subject measure of variability, defined as the difference in $\bar{\phi }$ between subjects, averaged over all unique combinations of paired subjects. To measure the effect of replacing territory‐specific phase offsets $\left(\phi_{i},\;i\subset \left\{1,\;2,\;3,\;4\right\}\right)$ with $\bar{\phi }$, i.e. a global phase offset approximation, the following theoretical result was derived from the modified Fermi function line shape (line shapes shown in Supporting Information Figure S1). In quantitative terms, using a global phase offset in place of a territory‐specific value causes 5% underestimation of Δ*M* if it is out by $14^{\circ }$ (assuming the preclinical line shape parameter values), and 10% underestimation of Δ*M* occurs if the difference is $22^{\circ }$. For the line shape values used with the human data, the equivalent thresholds are $13^{\circ }$ and $20^{\circ }$.

Velocity estimation was applied to the animal data and the estimated velocities were compared to the ground truth Doppler ultrasound measurements of carotid blood flow velocity using the Pearson correlation coefficient (*r*) and the root mean square (RMS) error. In the human data, the perfusion measures obtained using the standard analysis method, and bias‐corrected analysis (with and without velocity estimation), were compared. The analyses were repeated within subject‐specific high variability regions, defined as voxels where, in the acquired MP PCASL time series, the signal deviated more than 10% from the time series mean. In a previous study on healthy volunteers, these regions have been shown to correspond well with regions of hyperperfusion related to poor performance of the standard modified Fermi fitting algorithm.^26^ All results are presented as mean±standard deviation unless otherwise stated. The statistical measure for comparing quantitative perfusion was a repeated measures ANOVA test (2‐factor for the animal data, single‐factor for the human data), followed by Tukey's Honest Significant Difference post hoc test where applicable.

## 4 RESULTS

### 4.1 *In‐silico* comparison of standard and bias‐corrected analyses

Figure 2 illustrates the extent of bias observed when fitting a modified Fermi function to multi‐phase PCASL data using simulated data. As the SNR decreases there is a systematic over‐estimation bias of up to 20% introduced in the fitted parameters, which would translate to an error in perfusion at typical SNR. The bias seen in Figure 2A occurs across a range of SNR and magnitude values in Figure 2B and is particularly acute (15‐20%) at typical SNRs below 40, and at magnitudes that are 1‐4% of $M_{s}$. Figure 2C shows how the presence of bias is significantly reduced when a good estimate of ϕ is available. At an SNR of 10 and for a ratio $\frac{\Delta M}{M_{s}}$ of 1%, the estimation error decreased from 17.4% using a low‐precision estimate, to 6.46% when a high‐precision (constant) estimate of ϕ was used.

### 4.2 Variability of territorial phase offsets within, and between, subjects

**Figure 3:**
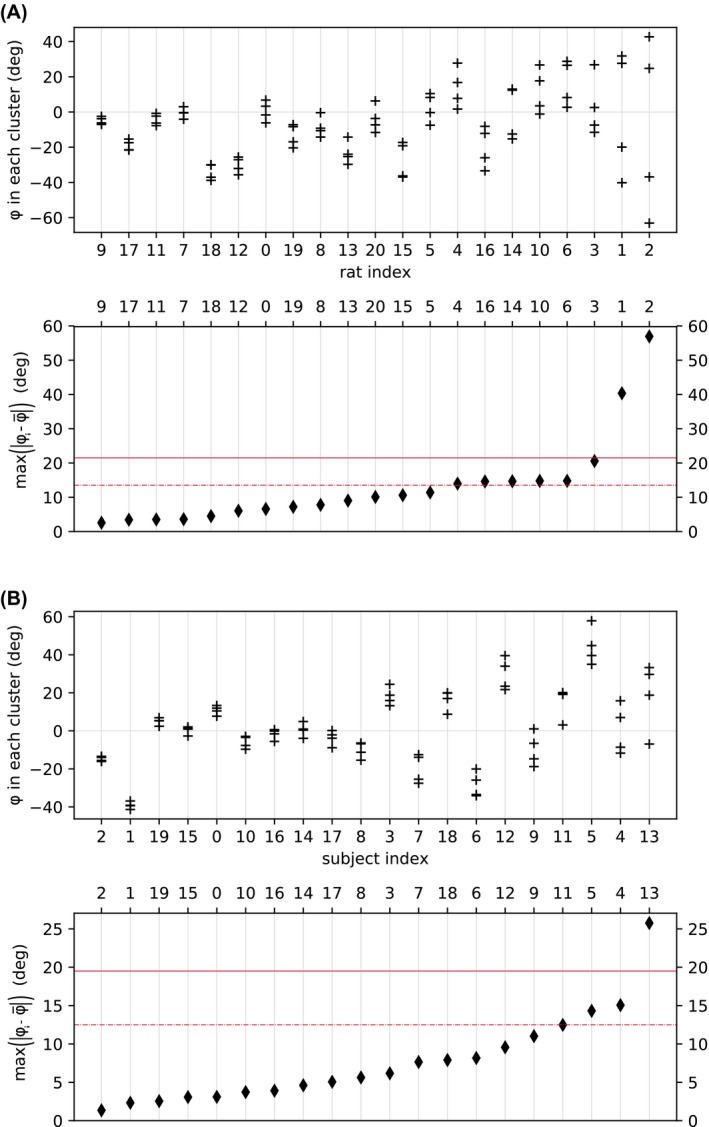
Estimated phase offsets (+ markers) in perfusion territories for (a) rats and (b) human subjects. The diamonds indicate how well a whole‐brain phase offset ($\bar{\phi }$) would approximate territory‐specific values ($\phi_{i}$). Diamonds higher than the red lines are subjects in which this approximation would incur an error in Δ*M* of at least 5% (dotted red), or least 10% (solid red)

The differences in estimated phase offset within subjects and between subjects are shown in Figure 3. Within subjects, the average difference between phase offsets was $14.1\;\pm \;14.1^{\circ }$ in rats, and $7.2\;\pm \;5.1^{\circ }$ in humans. There were 8 cases where phase clusters in rats were sufficiently disparate that forgoing cluster‐wise estimation, and using a whole‐brain phase approximation, would have incurred at least 5% error in estimation of Δ*M*. In human subjects, there were 4 cases where a whole‐brain phase approximation would have incurred at least 5% error in Δ*M*. Between subjects, differences in phase offset were relatively large: $16.1\;\pm \;5.0^{\circ }$ in rats, and $21.1\;\pm \;8.1^{\circ }$ in humans.

### 4.3 Perfusion estimation from animal data

**Figure 4:**
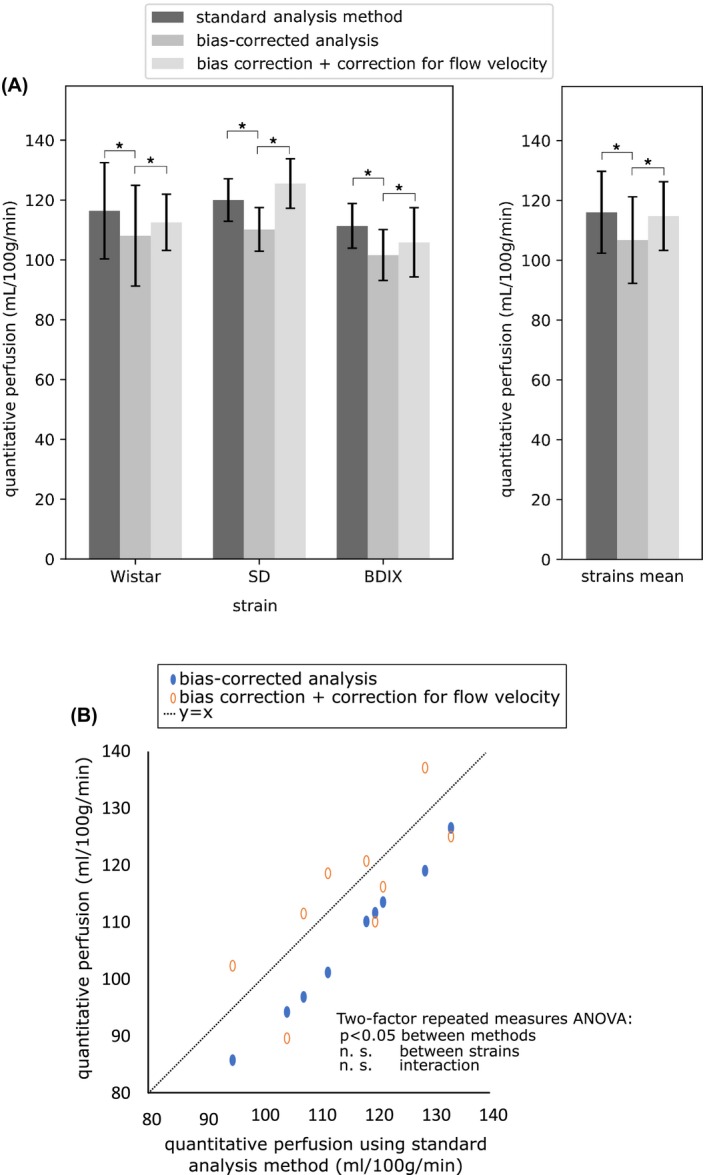
Perfusion estimated using standard analysis method, bias‐corrected analysis, and bias‐corrected analysis incorporating correction for flow velocity. A, Error bars are the standard deviation across rats. Asterisk indicates a significant pairwise difference in post hoc testing. B, Scatter plot of the corrected methods plotted against the standard analysis method

**Figure 5:**
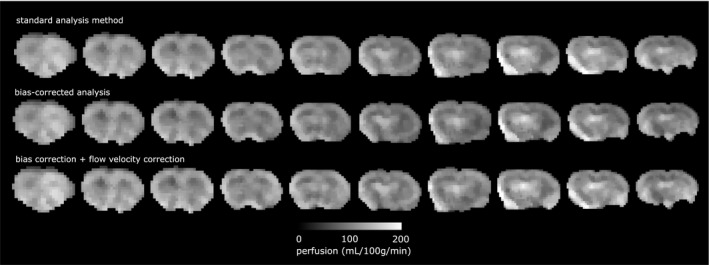
Slices from a Wistar rat comparing quantitative perfusion from the standard method of analysis, bias‐corrected analysis, and bias correction incorporating correction for flow velocity

Figure 4 compares the quantified perfusion in animal strains using the standard analysis approach, the bias‐corrected solution, and the bias‐corrected solution incorporating correction for flow velocity (slices of perfusion maps are shown in Figure 5). Across all strains (Wistar, SD, and BDIX respectively), the bias‐corrected measure of perfusion (108 ± 17, 110 ± 7, 102 ± 9 mL/100g/min) was lower than the standard analysis (116 ± 16, 120 ± 7, 111 ± 7 mL/100g/min), corresponding to a percentage decrease of 7.7%, 8.9%, and 9.6% for the respective strains. Across strains, the mean biased estimate was 116 ± 14 mL/100g/min, and the unbiased estimate was 8.0% lower, at 107 ± 14 mL/100g/min (*P* < 0.05). In order to relate this observed level of bias to the simulation data in Figure 2, the following parameters of the animal data were found: $\frac{\Delta M}{M_{s}}\;=\;2.1\;\pm \;0.3\%$, *SNR* = 63 ± 5. At this ratio of $\frac{\Delta M}{M_{s}}$ and SNR, the observed bias was consistent with the 6‐8% bias suggested by the simulation of multi‐phase data.

The modified Fermi function is only an approximation to the variation in signal inversion with phase offset. Simulations of profiles based on flow velocity might be more accurate in practice, and their inclusion in the multi‐stage solution is explored in terms of perfusion estimation and estimation of blood flow velocity. The coefficient of determination of the model fits was 0.96 ± 0.03 using the modified Fermi function, and 0.98 ± 0.01 using the velocity estimation model. When data were fitted using velocity estimation incorporated into the multi‐stage solution, the mean estimate of perfusion across strains was 115 ± 11 mL/100g/min. The difference between the bias‐corrected measures with and without velocity correction was 7.0% (*P* < 0.05).

**Figure 6:**
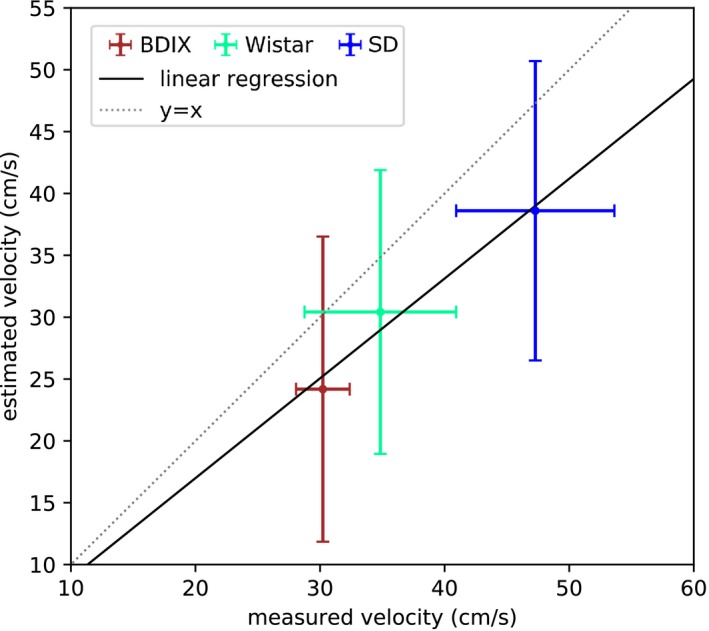
Scatter plot of mean blood flow velocity estimated in 3 rat strains using model fitting, compared to Doppler ultrasound measurements of mean carotid blood flow velocity. Error bars are the standard deviation across rats

The flow velocity estimates obtained from model fitting were correlated with Doppler ultrasound measurements of carotid blood flow velocity (*r* = 0.98, *P* = 0.12, linear regression: *y* = 0.81*x* + 0.01), as shown in Figure 6. The RMS error of the velocity estimates was 11.5 cm/s across the 3 strains. The estimated velocities were: 24.2 ± 12.3 cm/s for the BDIX strain (Doppler: 30.2 ± 2.2 cm/s); 30.4 ± 11.5 cm/s for the Wistar strain (Doppler: 34.8 ± 6.1 cm/s); and 38.6 ± 12.1 cm/s for the SD strain (Doppler: 47.3 ± 6.4 cm/s).

### 4.4 Perfusion estimation from human data

**Figure 7:**
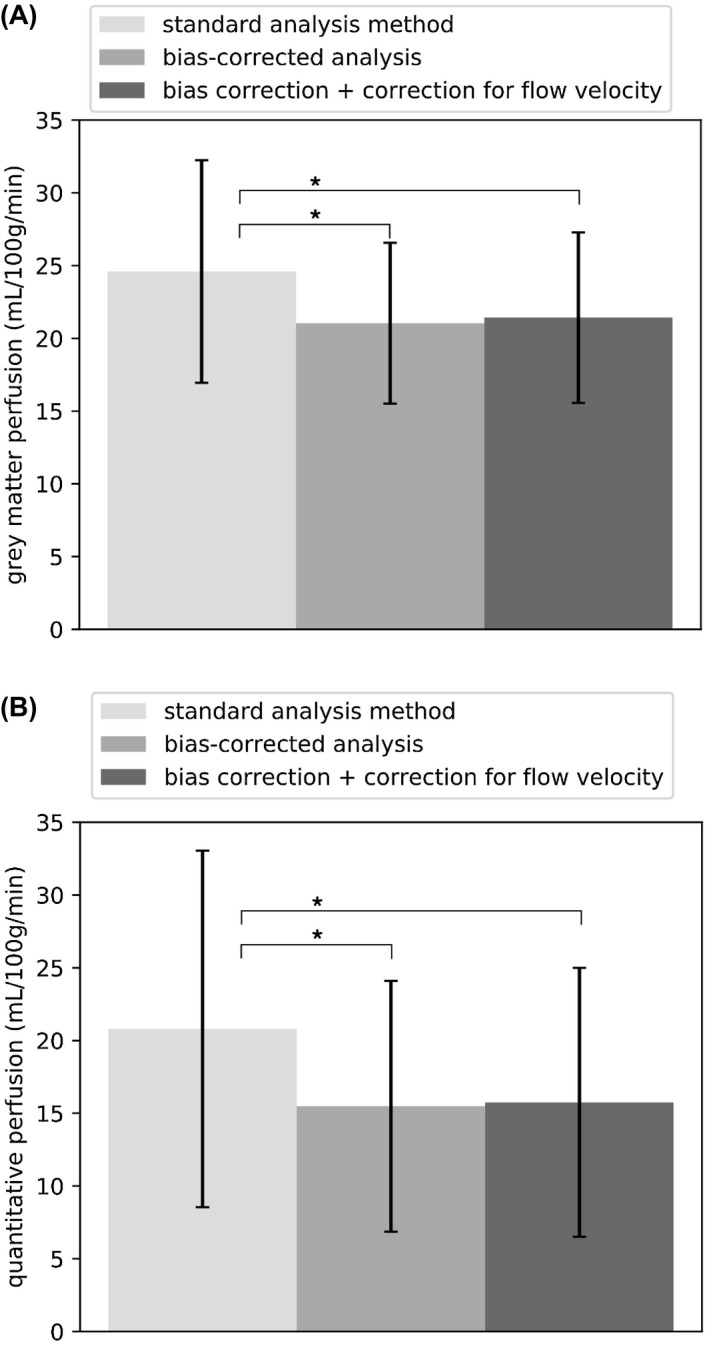
Perfusion estimated from human subject data using the standard analysis method, bias‐corrected analysis, and bias correction incorporating correction for flow velocity. A, Whole slice grey matter voxels, B, high variability voxels. Error bars are the standard deviation across subjects. Asterisk indicates a significant pairwise difference in post hoc testing

**Figure 8:**
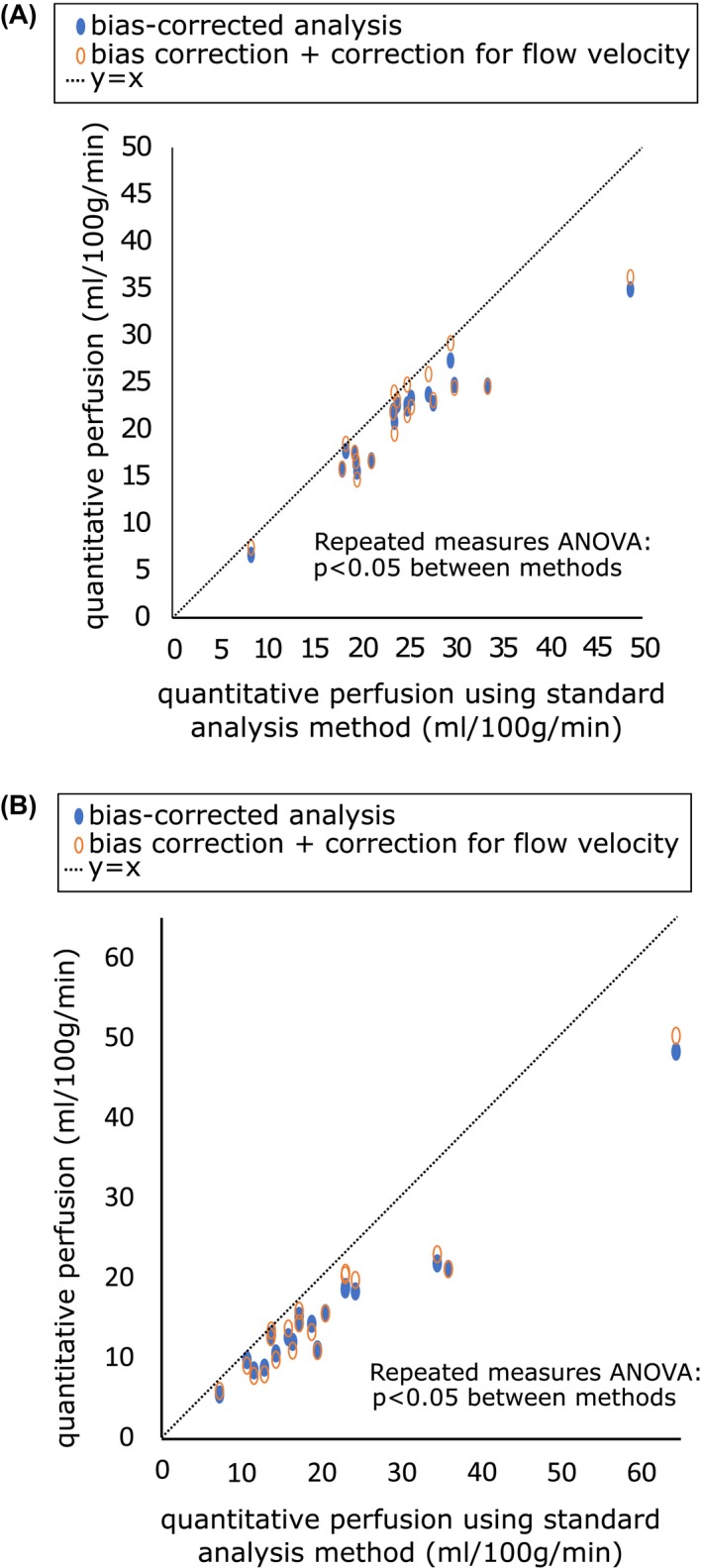
Scatter plots of the corrected methods plotted against the standard analysis method, shown for A, whole slice grey matter voxels, and B, high variability voxels

**Figure 9:**
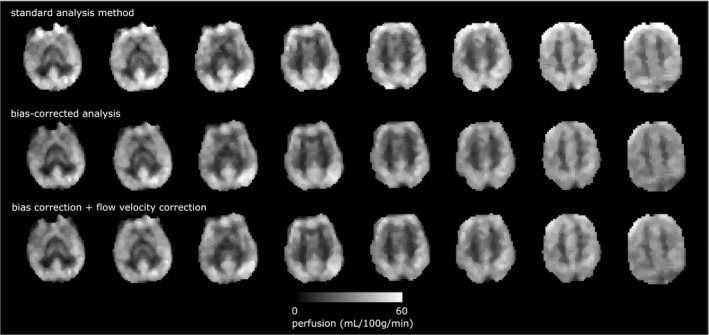
Slices from a human subject comparing quantitative perfusion from the standard method of analysis, bias‐corrected analysis, and bias correction incorporating correction for flow velocity

The coefficient of determination between the model fits and the acquired data was 0.91 ± 0.12 using the modified Fermi function, and 0.93 ± 0.10 using the velocity estimation model. The results of perfusion estimation using the standard analysis method, and using the bias‐corrected method, are compared in Figure 7, with the individual patient data points shown in Figure 8, and representative slices in shown in Figure 9A. Gray matter perfusion was 24.6 ± 7.7 mL/100g/min using the standard analysis method, and 21.0 ± 5.5 mL/100g/min using the bias‐corrected method, corresponding to a bias of 14.5% (*P* < 0.05). When correction for blood flow velocity was included, the perfusion measure was 21.4 ± 5.9 mL/100g/min. Within the high variability regions (Figure 9B), the values of quantitative perfusion obtained were: 20.8 ± 12.3 mL/100g/min using the standard analysis method, 15.5 ± 8.6 mL/100g/min using bias‐corrected analysis without correction for flow velocity, and 15.8 ± 9.2 mL/100g/min with correction for flow velocity. The corresponding decrease in estimated perfusion, with respect to biased analysis, was 25.5% without correction for flow velocity (*P* < 0.05), and 24.3% with correction for flow velocity (*P* < 0.05). The mean estimated velocity across subjects was 34 ± 11 cm/s.

## 5 DISCUSSION

Simulation of multi‐phase PCASL data has illustrated a recently reported over‐estimation bias in perfusion quantification from multi‐phase data.^9^ The accuracy of estimation of Δ*M* arising from model fitting was dependent upon the SNR of the data, but this was not purely a random effect as might be supposed. As the SNR decreased, a bias in the parameters of the model fit was observed. This has not previously been recognized for multi‐phase PCASL (prior to the recent study of Ref. [9]), but is consistent with studies that have been performed on the fitting of a sinusoidal profile to data with additive noise and a limited number of samples.^10^ An MP PCASL time series bears some similarity to a sinusoidal waveform, in that the following 4 parameters characterize some of its gross features: amplitude, phase shift, period, and amplitude offset. When estimating these 4 parameters, the bias is not in general positive.^10^ The condition under which estimation bias is positive is when the period is known, and where number of data points span an integer number of periods.^10^ These conditions are satisfied in MP PCASL as the time series index (representing RF phase offset) is expressed directly in radians, and the number of data points is chosen to cover a full period. In the absence of a good estimate of at least 1 model parameter, unbiased estimation of multi‐phase PCASL model parameters was not achieved with SNRs below 40, which is unlikely to be realized voxel‐wise from ASL data. To get an unbiased (or weakly biased) estimate, irrespective of SNR, it was only necessary to know 1 of 3 parameters, such as ϕ. When ϕ was known, the estimation of Δ*M* was markedly less dependent on the relative signal magnitude (the ratio of Δ*M* to the static tissue contribution). A low‐precision estimate of ϕ meant that small relative signal magnitudes, in the range of 1‐4%, exhibited pronounced bias, corresponding to quantification bias of 4‐16% for tissue perfusion.

The correction method implemented led to a reduction in bias that was similar in proportion across rat strains and in human data, and has also been successfully implemented in a recent preclinical study which sought to validate ASL perfusion measurements in rodents against gold‐standard autoradiography.^8^ The observed bias in the animal data of the present study was consistent with the expected bias suggested by the simulation of multi‐phase data. In the human subjects a low overall gray matter CBF was observed. Contributing factors are partial volume effects, inherently low perfusion expected in the geriatric population studied, combined with prolonged transit delays associated with aging that might lead to underestimation of perfusion given the PLD used. However, this does not detract from the change in perfusion seen with correction for the effects of bias in the multi‐phase fitting part of the analysis, which is consistent with that seen in simulations and the preclinical data.

Effects of blood flow velocity were incorporated into the multi‐stage solution by replacing the Fermi function with a more accurate model of the variation in the multi‐phase PCASL signal with phase offset that includes flow velocity. Velocity correction made a larger difference to quantitative perfusion in rats than in human subjects. In both cases, the quantitative difference made by velocity estimation was an increase in estimated perfusion. This may be because, in reference to Supporting Information Figure S1, the modified Fermi function overestimates the amplitude of the peak at typical flow velocities, and the degree of overestimation is greater for the preclinical PCASL parameters than it is for the human PCASL parameters. In the original MP PCASL study of Ref. [5], the parameters of the modified Fermi function (α = 54, β = 13) were based on a best RMS fit to 30 cm/s flow in human subject data. In the present study, the line shape (from the lookup table) that provided the optimal overall fit was indexed by a velocity of 34 cm/s, which is close to the 30 cm/s used by Jung et al However, the study by Jung et al used different PCASL tagging train parameters than those in this paper, thus the best RMS fit of the modified Fermi function might be slightly different for the current parameters, and this seems consistent with the slightly higher coefficient of determination obtained using the Bloch line shapes. On the other hand, the profiles in Supporting Information Figure S1B approximately agree with those in the Jung et al paper, even with different simulations approaches and scan parameters, so it seems that there is some robustness in the process. The simulations are for a single mean velocity and do not take into account cardiac pulsatility effects on the profiles, which might have different effects on the estimation process across physiological states, species and scan parameters.

**Figure 10:**
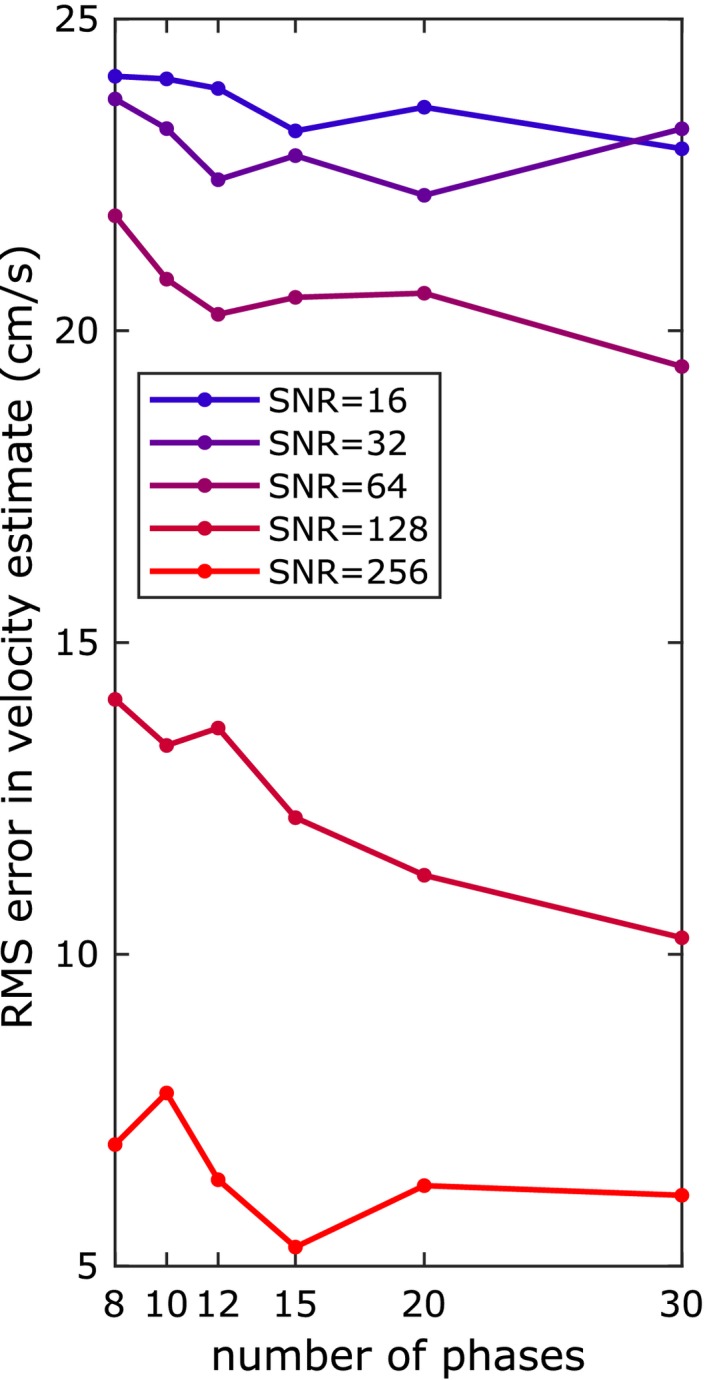
RMS error in velocity estimate from simulated multi‐phase PCASL data at different SNRs

The estimated blood flow velocities from the animal data were correlated with the velocity measurements from Doppler ultrasound, suggesting that the velocity estimation model consistently corrected for velocity‐dependent changes in the signal to within an error of 11 cm/s. To further explore the conditions under which blood flow velocity can be estimated, noisy multi‐phase PCASL data were generated from simulated velocity‐dependent inversion profiles constructed at different sampling densities (8 to 30 phases), to which Gaussian white noise was added at different SNRs, with the results shown in Figure 10. At the lower end of the SNR range (*SNR* ≤ 32), the velocity estimation error was approximately independent of the number of phases. At an SNR of 64, equivalent to the animal data used in this study, velocity estimation error using 8 phases was 22 cm/s and improved by only 2 cm/s for a 4‐fold increase in the number of phases. A doubling of SNR, on the other hand, reduced the estimation error by approximately 36%, indicating that the primary limiting factor in flow velocity estimation is SNR. The RMS error in velocity estimation observed the preclinical data suggests that the effective increase in SNR achieved by the multi‐stage algorithm was approximately 3‐fold over the voxel‐wise approach.

Generally the occipital vascular territory is perfused by the 2 vertebral arteries, as the latter fuse to form the basilar artery, thus blood supply can mix after labeling. In such a case, where voxels are supplied by both vertebral arteries, the solution we have proposed would not be optimal. This is because the modeling assumes a single off‐resonance source for each voxel. The general applicability of the model to the occipital vascular territory depends on how often we get mixed supply *and* have different phase offsets that are large enough not be well approximated by fitting only a single profile. In cases where the difference in phase offset between arteries is not very large, the modeling may still provide a reasonable approximation. This case of intra‐voxel mixing is partly a spatial resolution limit, and the only way to avoid it would be to use vessel‐encoded (VE) ASL.

In cases where the off‐resonance differences between territories are small, averaging over all voxels might provide similar perfusion results to using clustering. The nature of the modified Fermi function is that the measured Δ*M* is maximal when off‐resonance is correctly estimated, and Δ*M* is underestimated in all other cases. Therefore, the averaging approach would lead to a systematic underestimation of Δ*M* as it substitutes a whole‐brain average for territory‐specific off‐resonances. Within human subjects, estimated phase was generally consistent between perfusion territories, suggesting that phase offsets between territories are in most cases small enough to be well approximated by a single value, i.e. by fitting for a whole‐brain phase offset rather than a territory‐wise scheme. There are, however, some cases where clusters are different, such as the latter 4 subjects in Figure 3B, and in these instances a global phase offset may significantly underestimate perfusion in outlier clusters. Between subjects, differences in phase offset were relatively large, indicating that inter‐subject correction for phase offset is important.

An approximation to the clustering method, and one which might convey similar benefits, is to estimate the phase offset is estimated from a spatially blurred image, followed by estimation of Δ*M* from the unblurred image. There are 2 main ways in which simple spatial blurring and our proposed method differ in their ability to accurately estimate perfusion parameters. Simple blurring achieves a higher‐SNR estimate of voxel‐wise phase offset compared to when phase offset is estimated from an unblurred image. Greater degrees of spatial blurring afford higher SNR estimates of phase offset, though this trades off spatial resolution. The clustering method aims to strike a balance between maximizing the SNR for estimating phase offset, and preserving the cluster‐like spatial distribution of phase offset. High SNR is required for estimation to be robust to noise, and adequate spatial resolution is required for phase correction to be locally accurate. Reasoning on an anatomical basis, we have limited spatial resolution a priori to 4 clusters, and have proceeded to maximize SNR within this constraint by averaging all voxels within a cluster. SNR is thus maximized to the extent dictated by the spatial distribution of the data. At the boundary between phase offset regions, simple spatial averaging would mix phase offsets such that Δ*M* becomes underestimated. This could manifest as an artifact of low perfusion that traces the boundary between adjoining territories. This artifact is avoided in our method by clustering voxels before performing averaging, as clustering isolates voxels of different territories. A key point is that the clustering method will be just as good as simpler techniques, but in the cases where a simple method would fail, the clustering method would also cope. Thus our scheme is robust, even if the need to be robust is not very common.

The ROIs obtained from cluster analysis in this study would be expected to correspond approximately to the perfusion territories supplied by each of the arteries in the labeling plane. While there is a fair degree of consistency in these in both humans and rodents, the boundaries of these territories cannot be guaranteed, especially in pathology. While the boundaries of perfusion territories could be assessed using VE PCASL, this would largely defeat the object of using multi‐phase PCASL, essentially replacing it with a longer duration scan. In principle the clusters generated as part of the multi‐stage procedure could offer information about individual flow territories, although this would require further validation against vessel‐selective ASL imaging. Estimation of velocity could then allow for territory‐specific correction for labeling efficiency without needing artery selective ASL acquisition.

## 6 CONCLUSION

The observed bias in perfusion estimation from multi‐phase PCASL in animals was consistent with the expected bias suggested by simulation. By adopting a multi‐stage procedure incorporating an automated clustering to generate high SNR ROIs, a bias in perfusion quantification using multi‐phase PCASL data has been addressed. Effects of flow velocity were incorporated, enabling correction for flow‐dependent inversion efficiency as well as providing estimates of blood flow velocity that were correlated with ultrasound measurements.

## Supporting information


**FIGURE S1** The modified Fermi function (black) and Bloch simulation line shapes (colored), shown for A, the preclinical PCASL tagging parameters, and B, human subject PCASL parameters
