# Supplementary material for: Robust estimation of quantitative perfusion from multi‐phase pseudo‐continuous arterial spin labeling
Source: Magn Reson Med. 2019 Aug 20;83(3):815–29. doi: 10.1002/mrm.27965 (PMC6899553; doi:10.1002/mrm.27965)
Supplement: Supplementary file 1 — FIGURE S1 The modified Fermi function (black) and Bloch simulation line shapes (colored), shown for A, the preclinical PCASL tagging parameters, and B, human subject PCASL parameters [file MRM-83-815-s001.pdf]

Supporting Information

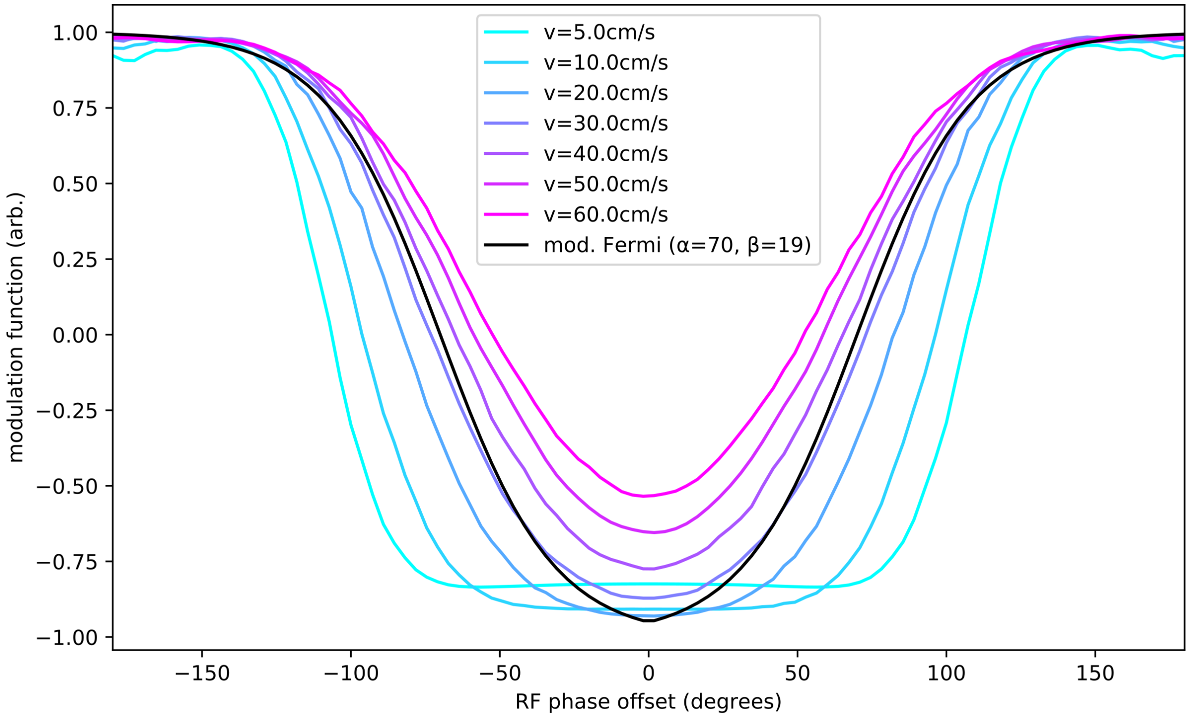

(a)

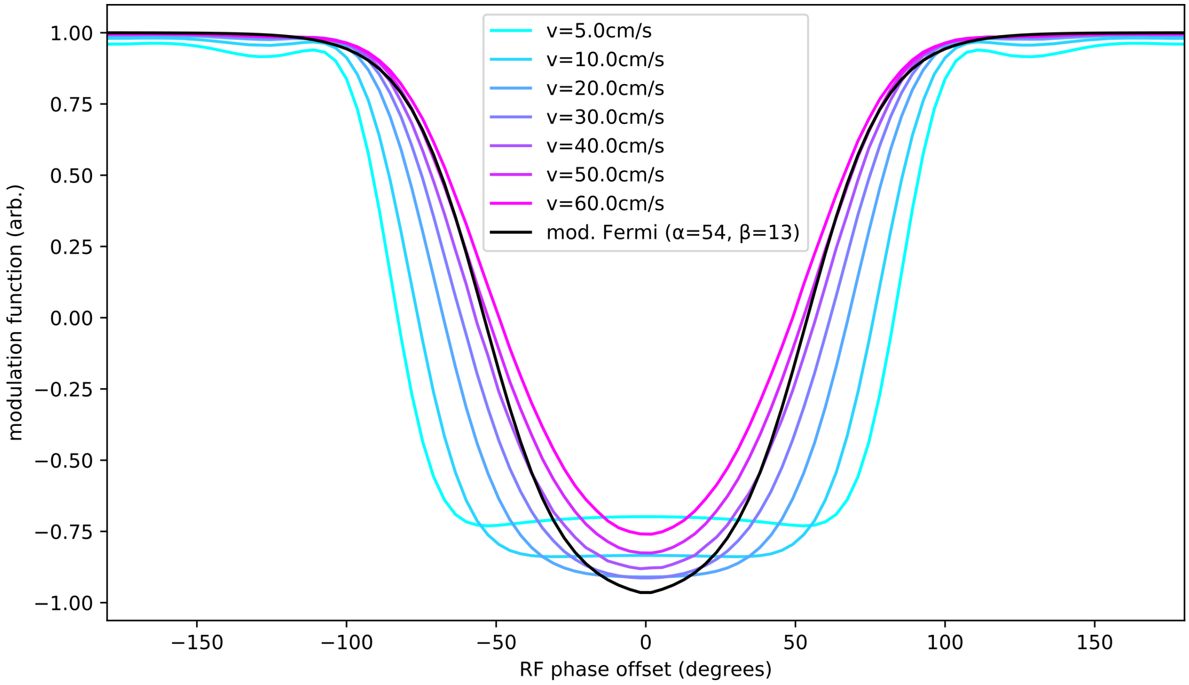

(b)

Supporting Information Figure S1: The modified Fermi function (black) and Bloch simulation line shapes (coloured), shown for (a) the preclinical PCASL tagging parameters, and (b) human subject PCASL parameters.
